# Barrett's esophagus transits to a cancer condition via potential biomarkers 

**Published:** 2018

**Authors:** Mohammad Reza Zali, Mohammad-Mahdi Zadeh-Esmaeel, Mostafa Rezaei-Tavirani, Elmira Sadat Tabatabaei, Nayeb Ali Ahmadi

**Affiliations:** 1 *Gastroenterology and Liver Diseases Research Center, Research Institute for Gastroenterology and Liver Diseases, Shahid Beheshti University of Medical Sciences, Tehran, Iran*; 2 *Skin Research Center, Shahid Beheshti University of Medical Sciences, Tehran, Iran*; 3 *Proteomics Research Center, Faculty of Paramedical Sciences, Shahid Beheshti University of Medical Sciences, Tehran, Iran*; 4 *Foodborne and Waterborne Diseases Research Center, Research Institute for Gastroenterology and Liver Diseases, Shahid Beheshti University of Medical Sciences, Tehran, Iran *

**Keywords:** Barrett's esophagus, Transcriptome, Protein interaction maps, Cancer development

## Abstract

**Aim:**

In this study, the transcriptome profile of Barrett's esophagus (BE) was examined for identification potential related biomarkers in view of interacting charactering.

**Background:**

Since BE is known as a precursor of esophageal cancer, the molecular studies of this condition could be essential.

**Methods:**

Gene expression data of BE in comparison with normal cases, GSE34619 was retrieved from Gene Expression Omnibus. Differentially expressed genes (DEGs) were determined applying GEO2R online software. The DEGs then were analyzed in terms of centrality properties via constructing an interaction network.

**Results:**

The data indicate that there are two sets of hub-bottlenecks panels with distinguishable values in BE. The first group shows that BE is very susceptible to develop cancer, and the second one implied on central characteristic of some DEGs as previously were also reported for BE pathogenicity. In addition, these genes are also implicated in cancer shift from certain conditions.

**Conclusion:**

On the whole, taking together these findings explain and support the cancerous origin of BE and introduced a panel of nominated biomarkers that could be more specific for BE rather than other types of esophageal problems. However, a complementary study to support this claim is suggested.

## Introduction

 Barrett's esophagus (BE) occurs in the distal esophagus in which the normal tissue is replaced by metaplastic columnar epithelium (1). About 1%–2% of the general population are affected by this condition that is progressed from gastro-esophageal reflux (GER). In addition, the incident of this condition is developing in western countries (2). Risk factors related to BE are anatomical (hiatus hernia), genetic, and lifestyle (smoking, consumption of alcoholic beverages, and hyperacidity (3, 4). One of the vital concerns related to BE is its capability to develop to Esophageal adenocarcinoma (EAC) as one of the important gastrointestinal cancers (5) with the chance of conversion of about 0.3% per year (3). While this chance is lower in BE without dysplasia, BE cases with dysplasia have the higher chance of developing EAC. Moreover, as the grade of dysplasia increases, the risk of developing to EAC grows as well which could be up to 10% yearly (1). As these sequence modification could be concluded to cancer state, it is important to establish novel detection methods for different types of BE with high sensitivity and specificity. For this aim, molecular studies are encouraged for identifying biomarkers with diagnosis and screening features (1). These investigations could be more beneficial if be studied in a high throughput format such as omics approaches. In this way, a set of identified biomarkers could be applicable for a disease surveillance and treatment approaches. Bioinformatics on the other hand, could provide more knowledge that is substantial in this regard. In a way that, introduced biomarkers by studies such as genomics, proteomics, and metabolomics can be further validated through bioinformatics (6). Those significant biomarkers in the disease state that have also central properties in an interaction network pattern of proteins, could be more promising relative to other ones (7). Therefore, protein-protein interaction (PPI) network analysis as a relatively new discipline may present additional concept of possible contributing markers of that specific disease (8). Here, putative biomarkers of BE are examined via analyzing and screening protein interaction maps and the related tactics to better understand the disease mechanisms and consequently applicable for intervention and treatment goals. 

## Methods

Microarray data series GSE34619 were obtained from GEO database which were included 10 differential gene expression profiles of BE and 8 normal squamous esophagus (NE) samples. Extracted RNAs from endoscopic samples were analyzed via GPL6244 platform. Data are published by di pietro M and coauthors (2012) entitled evidence for a functional role of epigenetically regulated midcluster HOXB genes in the development of Barrett's esophagus.

The profile samples were compared by boxplot analysis and to be considered as matched data via GEO2R analysis and the top 250 DEGs based on p-value were selected. Using p-value less than 0.05, 2≤ fold change (FC) ≤ 0.5, and excluding the uncharacterized individuals, the identified DEGs were included in PPI network analysis. The query genes were interacted by Cytoscape software (9) via STRING plugin. The network was analyzed by Network Analyzer application of Cytoscape software. Two central parameters including degree and betweenness centrality were considered to screen nodes of the network. The nodes with degree value above mean + 2 standard deviation were determined as hubs and the top 5% nodes based on betweenness were identified as bottlenecks (10). Common hubs and bottlenecks were introduced as hub-bottlenecks (central nodes). 

## Results

**Figure 1:**
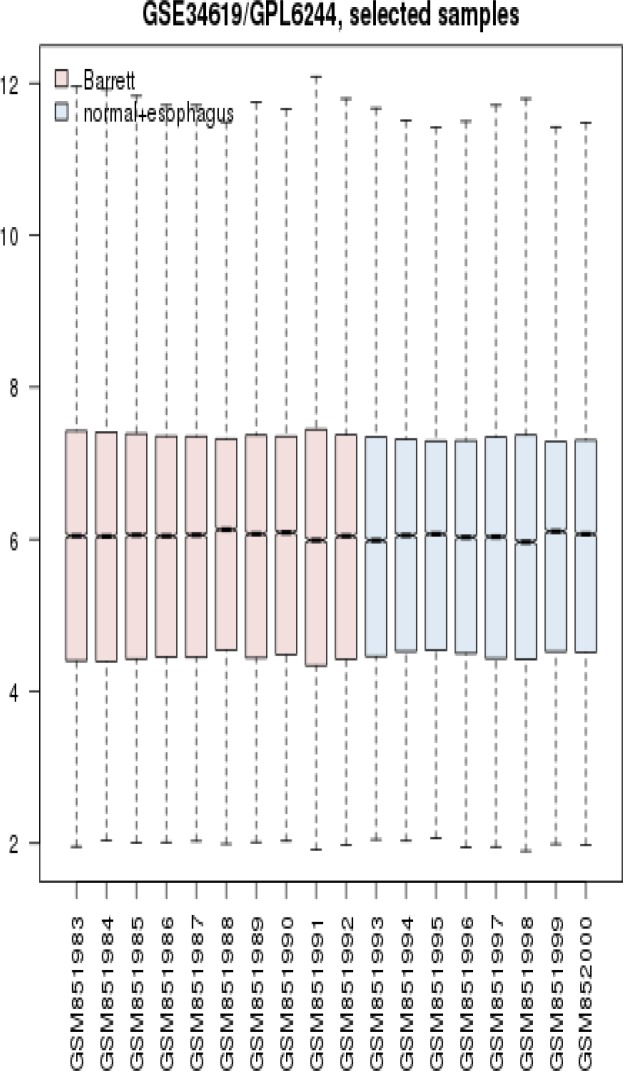
boxplot analysis of gene expression profiles 10 Barrett patients (red colored ones) and 8 normal-esophageal samples (blue colored samples) are presented. Vertical axe refers to normalized amount of gene expression change amounts and horizontal axe corresponds to samples

Since boxplot analysis is a suitable method which can be used to determine comparable samples to match profiles. As it is shown in figure 1 the samples are statistically comparable. Normalized distribution of gene expression profiles of Barrett patients and normal-esophageal individuals show similar pattern; however, include different DEGs.

**Figure 2:**
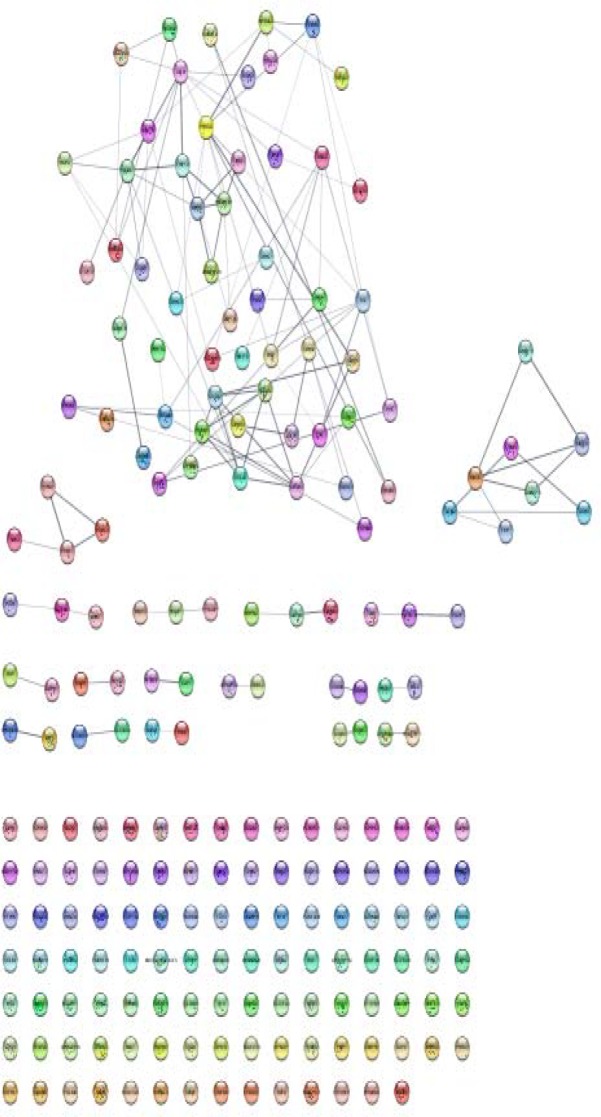
PPI network of 232 query DEGs of BE in comparison with NE is constructed by Cytoscape software via STRING database. 20 genes were not recognized ant the network includes: 110 isolated node, 11 double nodes, 4 triple, 1 tetrad, 1 8-nodes component, and a main connected component including 82 nodes and 96 edges

**Table 1:** Numbers of eight central nodes of Barrett network are presented. The nodes are sorted by BC value

R	name	description	Degree	BC
1	AKT1	v-akt murine thymoma viral oncogene homolog 1	110	0.0668
2	RHOA	ras homolog family member A	94	0.0482
3	PRDM10	PR domain containing 10	83	0.0461
4	SRC	v-src sarcoma (Schmidt-Ruppin A-2) viral oncogene homolog (avian)	85	0.0439
5	EGFR	epidermal growth factor receptor	82	0.0417
6	TP53	tumor protein p53	74	0.0403
7	HRAS	v-Ha-ras Harvey rat sarcoma viral oncogene homolog	77	0.0410
8	EGF	epidermal growth factor	77	0.0327

**Table 2:** **. **Numbers of eight central nodes of Barrett network (merely including the query genes) are presented. The nodes are sorted by BC value

R	name	description	Degree	BC
1	CFTR	cystic fibrosis transmembrane conductance regulator (ATP-binding cassette sub-family C, member 7)	51	0.02
2	PRKCA	protein kinase C, alpha	83	0.02
3	PPARG	peroxisome proliferator-activated receptor gamma	41	0.01
4	SH3GL1	SH3-domain GRB2-like 1	27	0.01
5	CXCR2	chemokine (C-X-C motif) receptor 2	53	0.01
6	LPAR3	lysophosphatidic acid receptor 3	49	0.01
7	GNA15	guanine nucleotide binding protein (G protein), alpha 15 (Gq class)	56	0.01
8	CCL28	chemokine (C-C motif) ligand 28	43	0.01

Numbers of 232 DEGs were included in PPI network analysis which 212 of them were recognized. The network was constructed and 128 connected components were identified (see figure 2). The network included 110 isolated nodes and 1 main connected component that was characterized by 82 nodes and 96 edges. As it is represented in the figure 2 the nodes of the constructed network do not have potent affinity to interact to each other’s. Numbers of 100 relevant genes were added to the query genes which led to construction of a main connected component including 247 nodes and 3584 edges. Degree value distribution equation (y=12.233x^-0.492^; correlation=0.852; R-squared=0.340 (which is computed on logarithmized values)) refers to scale free network. Numbers of eight central nodes were determined that are tabulated in the table 1. Since none of the query genes are not included in the central nodes, the eight hub-bottleneck genes including the query genes were introduced (see table 2).

## Discussion

The molecular study of BE has been in great attention recently. One of which is transcriptome profiling of normal subjects versus Barrett’s patients which is based on t-Test statistical analysis and evaluating the fold change of expression difference of genes. In this study the DEGs between these two conditions has been examined in terms of interaction properties. In the basic differential expression profiling, genes are introduced just by their importance in the expression changes; however, in a network analysis approach, the genes are screened and the most critical individuals can be recognized as the key genes of that disease. To get an informative examination of a network, some genes are required to participate as highly interacting elements. Otherwise, the network could not be considered as a scale-free network. In our study, at the first estimation of DEGs network construction, genes were not able to communicate with each other densely as it is represented in figure 2. Nevertheless, after adding neighbor genes to this constructed network of query genes, an informative pattern of an interacting network obtained that was valuable for continuing further analysis. Based on this finding, central nodes were identified as AKT1, RHOA, PRDM10, SRC, EGFR, TP53, HRAS, and EGF. Which all of them were from the neighbor genes known as added ones. In the other words, these genes can be characterized as elements that were essential in the network construction and foundation as well as integrity and strength of the network. Although these query DEGs are important based on significantly expression modification, still it is also important to examine their possible central role in the network. For this reason, on spite of considering the first 8 hub-bottlenecks that were from added genes we studied the next first 8 hub-bottlenecks that were among the query genes. These genes are CFTR, PRKCA, PPARG, SH3GL1, CXCR2, LPAR3, GNA15, and CCL28. Therefore, two panels of 8 elements associated with BE were introduced that in the first panel only centrality properties are considered but in the second one has the both centrality and expression values. In the first panel, genes are mostly relate to cancer and cellular cycle, cell proliferation, and cell signaling processes (11, 12). Two interpretations can be concluded from this group of identified central genes: first, these genes are mostly common among different malignancies. Second, these genes cannot be introduced as specific potential biomarkers of BE. Thus, the first concept can be interpreted as the relationship between BE and cancer. This indicated that BE could be regards as an Esophagus cancer risk factor as reported by some studies (13, 14). Hence, this is another support for these previous studies. 

Considering the second panel the top central DEGs is CFTR that has been previously reported to have some associations with BE and oesophageal adenocarcinoma (15). The second critical gene is PRKCA that is also involved in BE, esophagitis, esophageal squamous cell carcinoma, and adenocarcinoma of esophagus. It is also reported that PRKCA expression is associated with PLCE1(16). PPARG, the other element of this panel in addition to participate in Barrett's adenocarcinoma pathogenicity, plays crucial role in cell proliferation and apoptosis in different tumors as well(17). 

Moreover, there was no identified study related to involvement of SH3GL1, CXCR2, LPAR3, and GNA15in BE. Yet, CXCR genes are recognized by one study that is responsible for cancer development from esophagitis to a cancerous condition (18). Besides, LPAR and GNA15 genes showed some roles in cancer signaling for endometrial adenocarcinoma and esophagus tumor, respectively (19, 20). The latest gene, CCL28 demonstrated significant linkage in the progression from BE to the adenocarcinoma of esophagus (21). On the whole, all of the nodes of the panel 2, are linked to cancer development and consequently their altered expression may correspond to this incident. This fact may implies on the cancer oriented nature of BE and showing that which genes may have fundamental roles in this processes. Furthermore, the second panel in addition to its prominent feasible properties in cancer risk, could also be introduced as an exclusive set for BE (22). 

It can be concluded that there are some genes in BE network with possible responsibility to cancer condition transition. These genes are important to analysis and validate in large samples of interest for diagnosis and treatment goals.
